# Supplementary material for: From sequence to enzyme mechanism using multi-label machine learning
Source: BMC Bioinformatics. 2014 May 19;15:150. doi: 10.1186/1471-2105-15-150 (PMC4229970; doi:10.1186/1471-2105-15-150)
Supplement: Additional file 2 — Java code of ml2db. Additional file ml2db_code.tar.gz contains the Java source code to run the multi-label machine learning experiments and save the results to database. The code’s Javadoc is included. [file 1471-2105-15-150-S2.zip › additional file 2/ml2db/utils/doc/uk/ac/standrews/utils/main/database/package-tree.html]

uk.ac.standrews.utils.main.database Class Hierarchy


JavaScript is disabled on your browser.


- Overview
- Package
- Class
- Use
- Tree
- Deprecated
- Index
- Help

- Prev
- Next

- Frames
- No Frames

- All Classes

# Hierarchy For Package uk.ac.standrews.utils.main.database

Package Hierarchies:

- All Packages

## Class Hierarchy

- java.lang.Object
  - uk.ac.standrews.utils.main.database.DbConn (implements java.io.Serializable)
  - uk.ac.standrews.utils.main.database.DbConnectionSingleton
  - uk.ac.standrews.utils.main.database.DbManaged
    - uk.ac.standrews.utils.main.database.DbCreator
    - uk.ac.standrews.utils.main.database.DbReader
    - uk.ac.standrews.utils.main.database.DbWriter
  - uk.ac.standrews.utils.main.database.DbManagerSingleton
  - uk.ac.standrews.utils.main.database.DbUtils
  - uk.ac.standrews.utils.main.database.Managed
  - uk.ac.standrews.utils.main.database.Manager
    - uk.ac.standrews.utils.main.database.DbManager (implements java.io.Serializable)
      - uk.ac.standrews.utils.main.database.TableManager
        - uk.ac.standrews.utils.main.database.TableCreator
        - uk.ac.standrews.utils.main.database.TableReader
        - uk.ac.standrews.utils.main.database.TableRow
        - uk.ac.standrews.utils.main.database.TableWriter
  - uk.ac.standrews.utils.main.database.ResultSetUtils
  - uk.ac.standrews.utils.main.database.SqlUtils
  - uk.ac.standrews.utils.main.database.Table
  - uk.ac.standrews.utils.main.database.TableColumn (implements java.lang.Comparable<T>)

- Overview
- Package
- Class
- Use
- Tree
- Deprecated
- Index
- Help

- Prev
- Next

- Frames
- No Frames

- All Classes
